# Quantum Dots-Based Quantitative and In Situ Multiple Imaging on Ki67 and Cytokeratin to Improve Ki67 Assessment in Breast Cancer

**DOI:** 10.1371/journal.pone.0122734

**Published:** 2015-04-09

**Authors:** Jing Ping Yuan, Lin Wei Wang, Ai Ping Qu, Jia Mei Chen, Qing Ming Xiang, Chuang Chen, Sheng-Rong Sun, Dai-Wen Pang, Juan Liu, Yan Li

**Affiliations:** 1 Department of Oncology, Zhongnan Hospital of Wuhan University, Hubei Key Laboratory of Tumor Biological Behaviors and Hubei Cancer Clinical Study Center, Wuhan, Hubei, China; 2 School of Computer, Wuhan University, Wuhan, Hubei, China; 3 Department of Breast and Thyroid Surgery, Renmin Hospital of Wuhan University, Wuhan, Hubei, China; 4 Key Laboratory of Analytical Chemistry for Biology and Medicine (Ministry of Education), College of Chemistry and Molecular Sciences, State Key Laboratory of Virology, and Wuhan Institute of Biotechnology, Wuhan University, Wuhan, Hubei, China; University of Torino, ITALY

## Abstract

**Background:**

As a marker for tumor cell proliferation, Ki67 has important impacts on breast cancer (BC) prognosis. Although immunohistochemical staining is the current standard method, variations in analytical practice make it difficult for pathologists to manually measure Ki67 index. This study was to develop a fluorescent spectrum-based quantitative analysis of Ki67 expression by quantum-dots (QDs) multiple imaging technique.

**Methods:**

A QDs-based in situ multiple fluorescent imaging method was developed, which stained nuclear Ki67 as red signal and cytoplasmic cytokeratin (CK) as green signal. Both Ki67 and CK signals were automatically separated and quantified by professional spectrum analysis software. This technique was applied to tissue microarrays from 240 BC patients. Both Ki67 and CK values, and Ki67/CK ratio were obtained for each patient, and their prognostic value on 5-year disease free survival was assessed.

**Results:**

This method simultaneously stains nuclear Ki67 and cytoplasmic CK with clear signal contrast, making it easy for signal separation and quantification. The total fluorescent signal intensities of both Ki67 sum and CK sum were obtained, and Ki67/CK ratio calculated. Ki67 sum and Ki67/CK ratio were each attributed into two grades by X-tile software based on the best *P* value principle. Multivariate analysis showed Ki67 grade (*P* = 0.047) and Ki67/CK grade (*P* = 0.004) were independent prognostic factors. Furthermore, area under curve (AUC) of ROC analysis for Ki67/CK grade (AUC: 0.683, 95%CI: 0.613–0.752) was higher than Ki67 grade (AUC: 0.665, 95%CI: 0.596–0.734) and HER-2 gene (AUC: 0.586, 95%CI: 0.510–0.661), but lower than N stage (AUC: 0.760, 95%CI: 0.696–0.823) and histological grade (AUC: 0.756, 95%CI: 0.692–0.820) on predicting the risk for recurrence.

**Conclusions:**

A QDs-based quantitative and in situ multiple imaging on Ki67 and CK was developed to improve Ki67 assessment in BC, and Ki67/CK grade had better performance than Ki67 grade in predicting prognosis.

## Background

Breast cancer (BC) is the most common malignant tumor among females in the world [1,2]. It has been widely accepted by the oncology community that BC is a highly heterogeneous disease, with different biological behaviors for the same stage of BC among different patients [3]. As the result of continuous insights into the BC molecular biology, personalized therapy based on the different tumor molecular classifications has become the main direction in BC treatment [4]. The prerequisite for personalized medicine is the correct understanding of prognostic and predictive parameters, which remains an important and active research field in BC management. With the development of molecular bio-medicine, many BC-related molecules have been explored, and the most commonly used markers in clinical practice include human epidermal growth factor receptor-2 (HER2), estrogen receptor (ER), progesterone receptor (PR), and Ki67 [5,6]. Among them, Ki67, an antigen discovered in the early 1980s has been reported to have close relationship with the prognosis of BC [7–10].

**Fig 1:**
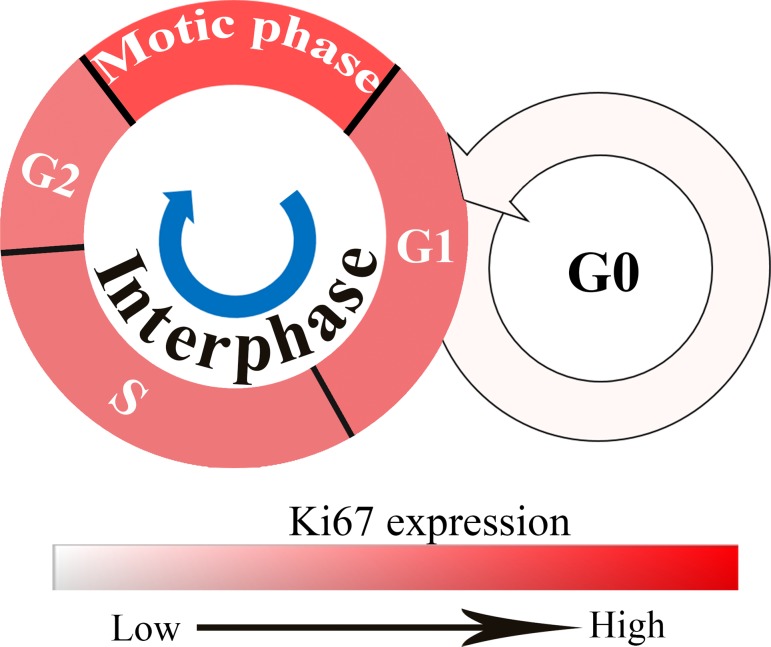
Ki67 expression in different cell cycle phases. Cells express Ki67 during G1, S, G2, and mitotic phases but not during G0. Ki67 levels are low in the G1 and S phases and rise to peak level in mitotic phase; the depth of red color represents Ki67 expression levels, the deeper the higher.

Ki67 is a nuclear antigen originally identified by Gerdes et al [11] in the early 1980s, by using a mouse monoclonal antibody against a nuclear antigen from a Hodgkin lymphoma-derived cell line. The antigen named after the researcher’s location, Ki for Kiel University, Germany, with the 67 label referring to the clone number on the 96-well plate [11]. Ki67 expression varies with different cell cycle phases. Cells express Ki67 during G1, S, G2, and mitotic phases, but not during the resting phase G0. Ki67 levels are low in G1 and S phases, but rise to a peak level in mitotic phase. In later periods (anaphase and telophase) of mitotic phase, Ki67 levels decrease sharply (Fig 1) [12]. As a wildly used proliferation marker, Ki67 has attracted increasing attention in recent years. Many studies showed that Ki67 expression levels were negatively correlated with BC prognosis [7,10,13–15]. However, it is difficult to manually measure the Ki67 expression accurately and objectively, significantly limiting its clinical applications [8,16]. Automated counting of Ki67 by computer software could be helpful, however, there is no evidence to show it is better than manual counting. And the results obtained by automated counting with traditional computer software is the sum of Ki67 expression, not the percentage of Ki67 which may be influenced by the number of cancer cells [17,18]. Thus, it is urgent to develop a more accurate and objective method to evaluate the Ki67 expression in BC.

Quantum dots (QDs) are fluorescent nanoparticles with unique size and surface effects which have been wildly used in bio-imaging, targeting and detecting due to their excellent properties, such as high fluorescence intensity, strong resistance to photobleaching and chemical degradation, size-tunable emission wavelength and simultaneous multiple fluorescent colors under single source excitation [19–22]. In this study, we aimed to establish a QDs-based quantitative and in situ multiple imaging on Ki67 and cytokeratin (CK, a kind of epithelial specific marker [23,24] used to label cancer cells in this study), so as to better evaluate their impacts on BC prognosis. In order to evaluate Ki67 expression more accurately and objectively, the Ki67 percentage was replaced by Ki67/CK ratio.

## Methods

### Patients

**Fig 2:**
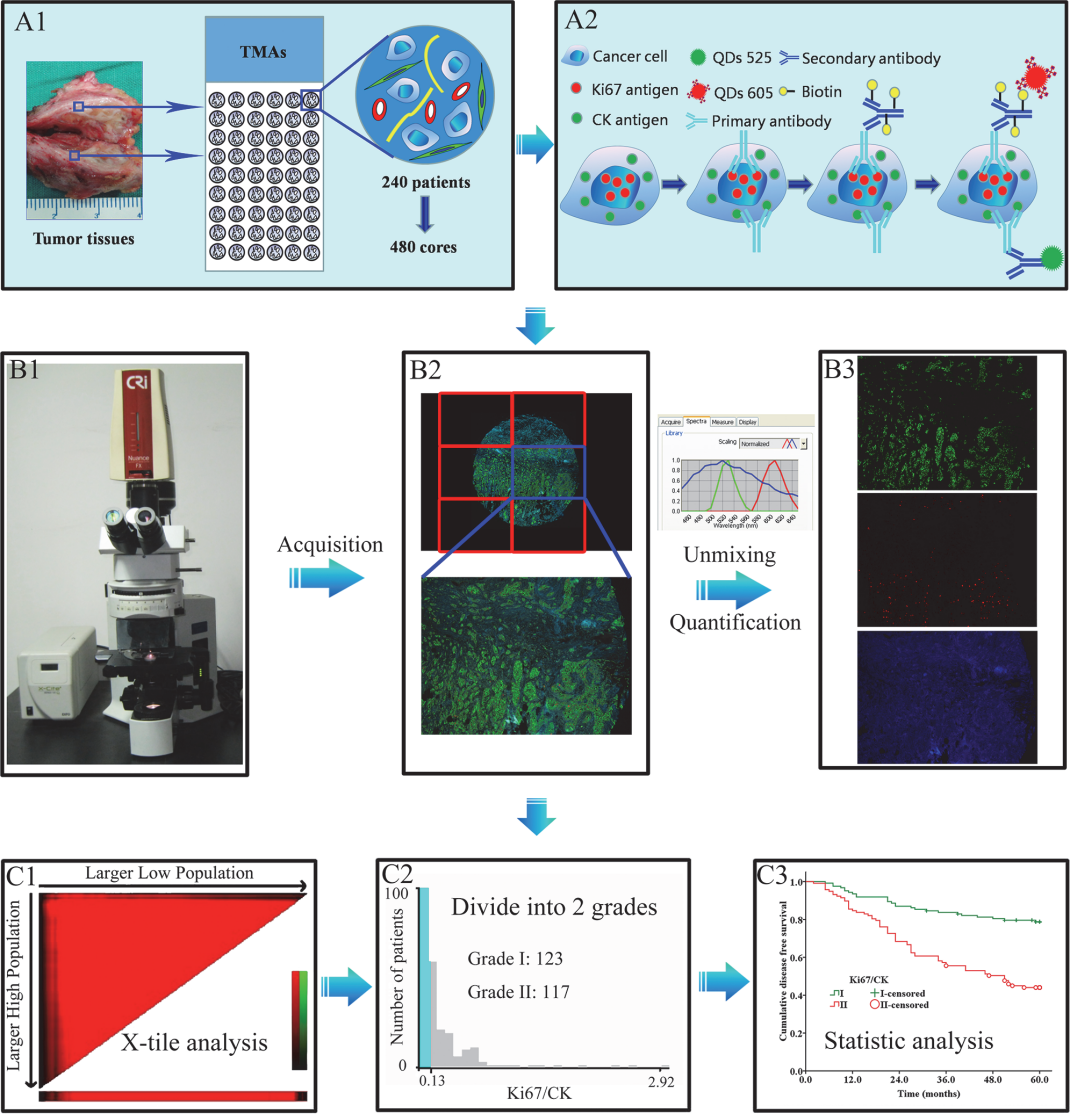
The design and major technical procedures of this study. Panel A1, 240 cases of BC specimens were constructed into TMAs with 480 cores. Panel A2, CK and Ki67 were stained with QDs multiple imaging method. Panels B1 → B3: images acquisition, unmixing and quantification by CRi Nuance software. Panels C1 → C3: Ki67 and Ki67/CK values were analyzed by X-tile software (C1), divided into two grades (C2), and analyzed by Kaplan-Meier method (C3). BC: breast cancer; QDs: quantum dots; TMAs: tissue microarrays; CK: cytokeratin.

A comprehensive BC database has been established at our cancer center. The database collection and tissue microarrays (TMAs) construction were based on clearly set criteria, and complete clinico-pathological information on the patients was available. The TMAs included 240 BC specimens (480 cores, 1.5 mm each core) with five years follow-up (Fig 2A1). Major clinico-pathological information including age, menopausal status, T stage, N stage, histological grade, ER and HER2 gene status was summarized in Table 1. TNM staging and histological grade were classified according to the 7th edition of American Joint Committee on Cancer (AJCC) TNM system [25] and the 4th edition of WHO histological grade [26]. As this work was based on our systematically established database, ER and HER2 status were determined by the expert pathologists in clinical laboratories based on the most updated pathological guidelines, immunohistochemical (IHC) for ER [27] and fluorescent in-situ hybridization (FISH) for HER2 [28]. The study protocol was approved by the Institutional Ethics Committee of Zhongnan Hospital of Wuhan University and was undertaken according to the ethical standards of the World Medical Association Declaration of Helsinki.

**Table 1:** Major clinico-pathological characteristics of 240 breast cancer patients.

**Items**	**n (%)**
**Age (years)**	
**≤ 50**	149 (62.1)
**> 50**	91 (37.9)
**Menopausal status**	
**Premenopausal**	134 (55.8)
**Postmenopausal**	106 (44.2)
**T stage (cm)**	
**T1 (T ≤ 2)**	36 (15.0)
**T2 (2 < T ≤ 5)**	162 (67.5)
**T3 (T > 2)**	42 (17.5)
**N stage**	
**N0**	109 (45.4)
**N1**	66 (27.5)
**N2**	30 (12.5)
**N3**	35 (14.6)
**Histological grade**	
**I**	40 (16.7)
**II**	141 (58.8)
**III**	59 (24.6)
**ER status** ^**a**^	
**Positive**	106 (44.2)
**Negative**	134 (55.8)
**HER2 gene** ^**b**^	
**Amplification**	51 (21.3)
**Non-amplification**	189 (78.7)

^a^ER was determined by immunohistochemical staining according to guideline [27];

^b^HER2 gene was determined by fluorescent in-situ hybridization (FISH) according to guideline [28].

T: tumor; N: node; ER: estrogen receptor; HER2: human epidermal growth factor receptor-2.

### QDs-based multiple imaging of CK and Ki67

QDs-based fluorescent multiple imaging technique has been established at our cancer center with detailed procedures reported previously [29–31] and briefed as the following (Fig 2A2). TMAs heating → de-paraffinizing → antigen retrieval → blocking → primary antibodies for Ki67 and CK → biotinylated IgG → staining with QDs-525 and QDs-605 simultaneously → washing → image acquisition and quantification. Ki67 immunostaining was performed using anti-Ki67 rabbit monoclonal antibody (Clone: SP6; dilution: 1:100). SP6 antibody has been confirmed to have equal specificity with MIB6 [17,32], but slightly better performance for quantitative image analysis [33]. Biotinylated anti-rabbit IgG (dilution: 1:400) and streptavidin conjugated QDs-605 (dilution: 1:600) were applied to develop the red fluorescence of Ki67 at cell nucleus. CK immunostaining was performed using anti-CK mouse monoclonal antibody (Clone: AE1/AE3; dilution: 1:100). QDs-525 conjugated goat anti-mouse IgG (dilution: 1:50) was applied to develop the green fluorescence of CK at the cytoplasm. The above antibodies and QDs were kindly provided by Wuhan Jiayuan Quantum Dots Co., Ltd. (Wuhan, China).

In order to validate the above imaging technique and compare the technical features of different imaging methods in BC, we conducted direct comparisons of three imaging methods, i.e. conventional hematoxylin and eosin (HE) staining, IHC staining of CK and Ki67 separately, and QDs-based multiple imaging of CK and Ki67 simultaneously. Serial tissue sections of breast invasive ductal carcinoma were stained by these methods. The HE staining and routine IHC staining followed the standard technical procedures in clinical pathology, and the agents and antibodies were the same as the above.

### Image acquisition, quantification and analysis

QDs fluorescence information on Ki67 and CK was simultaneously obtained based on 6 photographs for each TMAs core under Olympus BX51 fluorescent microscope (Olympus Optical Co., Ltd. Tokyo, Japan) equipped with CRi Nuance multispectral imaging system (Cambridge Research and Instrumentation, Inc., Woburn, MA, USA) at 100× magnifications. The images of 6 photographs were merged to form a panorama spectral image for each BC TMAs core. A spectral cube for each core of TMAs containing the complete spectral information at 10-nm wavelength intervals from 450 nm to 650 nm was collected by CRi Nuance multi-spectral imaging systems under the same exposure time (800 ms).

After imaging acquisition, the unmixing and quantification on Ki67 and CK were performed by the software package within CRi Nuance multispectral imaging system. There are 3 technical steps for this procedure: (1) Selection of targets with different spectra: Ki67, CK and tissue autofluorescence were selected as red (605 nm), green (525 nm) and blue signals (automatically set by the software), respectively (Fig 2B1–2B3); (2) Image unmixing and elimination of background noise: the target images with different spectra were automatically unmixed by the software based on specific spectrum into 3 separate images, i.e. CK image with green signal (Fig 2B3, upper panel), Ki67 image with red signal (Fig 2B3, middle panel), and blue background (Fig 2B3, lower panel) which was deleted at the last step of quantification; and (3) Target spectrum quantification: both CK and Ki67 spectral signals were automatically quantified by CRi Nuance system.

The original results produced by CRi Nuance software were total signals for Ki67 and CK in one view field. As each TMAs core has 6 view fields, the final results output was Ki67 sum and CK sum, respectively, i.e. signal pixels in 6 fields altogether. The Ki67 sum and CK sum were recorded one-by-one. To calculate the Ki67 signal proportion among the CK signal background, Ki67/CK ratio was defined as (Ki67 sum ÷ CK sum), representing the proportion of tumor cells at active proliferation among all the tumor cells in the TMAs core. As this computer-based signal acquisition method takes into consideration of all tumor cells rather than a portion of tumor cells (e.g. 1000 tumor cells as suggested by Dowsett et al [17]), it could help minimize errors in data interpretation and scoring and improve data quality.

Once the Ki67 sum and Ki67/CK ratio results were obtained, the X-tile software, developed by scholars at Yale University in 2004 to assess biomarkers and find optimal cut-point of biomarkers based on the outcome [34], was applied to automatically judge the cut-off points of Ki67 sum and Ki67/CK ratio, and divided them into two categories, designated as grade I and grade II according their values, respectively (Fig 2C1–2C3).

### Statistical analysis

Statistical analysis was performed with SPSS 17.0 software (SPSS Inc. Chicago, IL, USA). Correlation test was calculated by Pearson chi-square. Five-year disease free survival (5-DFS) was the primary end-point and determined by the Kaplan-Meier method and analyzed by the log-rank test. Multivariate analysis was performed by the Cox proportional hazards model. Receiver operating characteristic (ROC) curve analysis was used to calculate the predictive value of the independent prognosis factors. Two sided *P* < 0.05 was considered as statistically significant.

## Results

### Comparison between QDs-based multiple imaging and traditional staining

**Fig 3:**
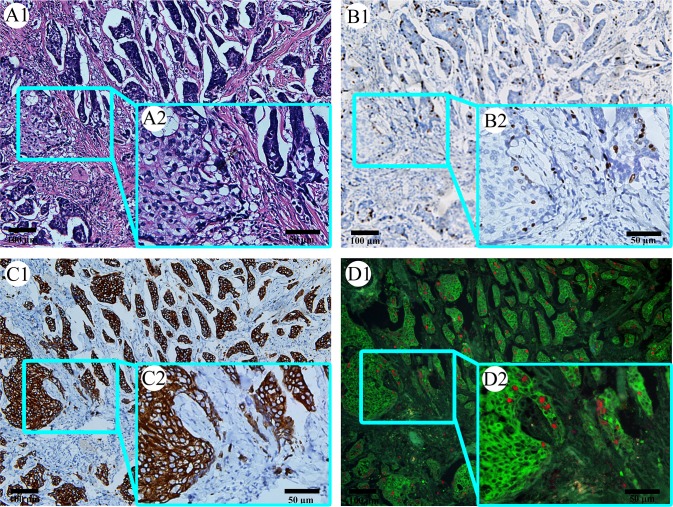
Comparison between QDs-based multiple imaging and traditional staining. HE staining can show histological structures, but not label Ki67 and tumor nests well (A1 and A2). Immunohistochemical staining on Ki67 only can label Ki67 clearly, but not on tumor nests well (B1 and B2). Immunohistochemical staining on CK can label tumor nests clearly, but not on Ki67 well (B1 and B2). QDs-based multiple imaging can simultaneously label Ki67 and tumor nests clearly (D1 and D2). (In each panel, image 2 is the magnification of the square part of image 1. Magnification: A1, B1, C1 and D1: 100×; A2, B2, C2 and D2: 200×). QDs: quantum dots; HE: hematoxylin and eosin; CK: cytokeratin.

Serial tissue sections of breast invasive ductal carcinoma were stained by HE staining, IHC staining of CK and Ki67 separately, and QDs-based multiple imaging of CK and Ki67 simultaneously, in order to directly compare the technical features of different imaging methods in BC (Fig 3). HE staining (Fig 3A1 and A2) can show the histological structures of tumor, with cancer cells compacted into tumor nests invading the stroma, but the invasion edge of tumor nests sometimes could not be clearly revealed by such technique, because there is no definite image differentiation between the tumor and the stroma. IHC staining of Ki67 (Fig 3B1 and 3B2) can only label Ki67 clearly, but could not clearly show tumor nests, rendering it technically difficult to precisely quantify the percentage of Ki67 in the tumor nests. Likewise, IHC staining of CK (Fig 3C1 and 3C2) can label tumor nests clearly, but could not label Ki67 well. In contrast, QDs-based multiple imaging on CK and Ki67 (Fig 3D1 and 3D2) can simultaneously label Ki67 and tumor nests well, with clear imaging differentiation between tumor nests and the stroma and between CK and Ki67. Therefore, this method makes it possible for spectrum-based accurate and quantitative analysis of CK and Ki67.

### Ki67 sum and Ki67/CK ratio quantification and grades

Detailed information on clinico-pathological features and Ki67 sum, CK sum, and Ki67/CK ratio for each patient was listed in S1 Dataset. The X-tile software based on the best *P* value principle was adopted to automatically judge the cut-off points of Ki67 sum and Ki67/CK ratio as 3.58×10^7^ and 0.186, using 5-DFS as a prognosticator. Subsequently, Ki67 sum and Ki67/CK ratio each was divided into two grades based on these cut-off points. Patients with Ki67 sum from 2.71×10^4^ to 3.58×10^7^ and better 5-DFS were classified into Ki67 grade I (n = 91), and those with Ki67 sum from >3.58×10^7^ to 2.88×10^8^ and poor 5-DFS were classified into Ki67 grade II (n = 149). Similarly, patients with Ki67/CK ratio from 1.95×10^−4^ to 0.186 and better 5-DFS were classified into Ki67/CK grade I (n = 123), and those with Ki67/CK ratio from >0.186 to 4.19 and poor 5-DFS were classified into Ki67/CK grade II (n = 117). Detailed information on such classification was listed in Table 2.

**Table 2:** Ki67 sum and Ki67/CK ratio quantification, cut-off points and grades.

Items	Results		Cut-off point of I/II	Cases in each grade (n)	
	Median	Range		I	II
**Ki67 sum**	2.30×10^7^	2.71×10^4^–2.88×10^8^	3.58×10^7^	91	149
**Ki67/CK ratio**	0.186	1.95×10^−4^–4.19	0.186	123	117

CK: cytokeratin.

### Patterns of Ki67 expression

**Fig 4:**
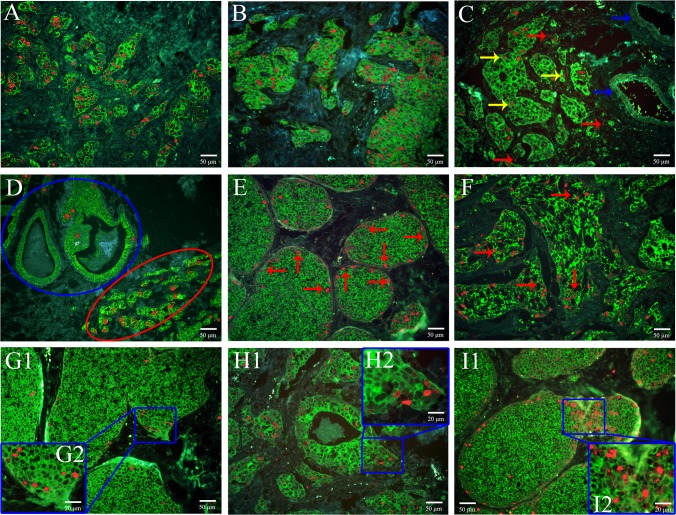
Various patterns of Ki67 expression in BC. Ki67 grade I and Ki67/CK grade II in BC tissues (A, 200 ×); Ki67 grade II and Ki67/CK grade I in BC tissues (B, 200 ×); Ki67 is mainly expressed in cancer cell nucleus (C, yellow arrows, 200 ×), with few exceptions in stroma (C, red arrows), not in blood vessel (C, blue arrows); Ki67 expression levels are higher in invasive cancer nests (D, red ellipse, 200 ×) than carcinoma in situ (D, blue ellipse); for carcinoma in situ, Ki67 expression is more prominent at the tumor nest periphery than at the center (E, red arrows, 200 ×); for invasive carcinoma, haphazard Ki67 expression is observed in the disorganized tumor nests (F, red arrows, 200 ×); high Ki67 expression is observed at the tumor nests invasion edges both for carcinoma in situ with minimal invasion (G1, 200 ×; G2, 400 ×) and invasive ductal carcinoma (H1, 200 ×; H2, 400 ×); when a big tumor nest produces smaller seeding tumor nests, high Ki67 expression is observed at the conjunction between the two tumor nests (I1, 200 ×; I2, 400 ×). BC: breast cancer; CK: cytokeratin.

During the TMAs images acquisition, attentions were also paid to the patterns of Ki67 expression in BC tissues. The following Ki67 expression patterns were observed (Fig 4). (1) Ki67 grade I and Ki67/CK grade II in BC tissues (Fig 4A). This means although the absolute Ki67 expression is low, the proportion of Ki67 positivity among the tumor tissue is high, indicating high tumor cell proliferation. (2) Ki67 grade II and Ki67/CK grade I in BC tissues (Fig 4B). This means although the absoluteKi67 expression is high, the proportion of Ki67 positivity among the tumor tissue is low, indicating low tumor cell proliferation. (3) Ki67 is mainly expressed in cancer cell nucleus (Fig 4C, yellow arrows), with few exceptions in stroma (Fig 4C, red arrows), but not in blood vessel (Fig 4C, blue arrows). This means Ki67 could be used as a specific marker indicating proliferating tumor cells. (4) Ki67 expression is higher in invasive ductal carcinoma nests (Fig 4D, red ellipse) than in situ carcinoma nests (Fig 4D, blue ellipse). This means that Ki67 expression could be directly related with tumor invasiveness. (5) For carcinoma in situ, Ki67 expression is more prominent at the tumor nest periphery than at the center (Fig 4E, red arrows). This reflects the expanding growth feature of carcinoma in situ. (6) For invasive carcinoma, haphazard Ki67 expression is observed in the disorganized tumor nests (Fig 4F, red arrows). This indicates the disordered tumor cell arrays with individual cancer cells competing for proliferating advantages. (7) High Ki67 expression is observed at the tumor nests invasion edges both for carcinoma in situ with minimal invasion (Fig 4G) and invasive ductal carcinoma (Fig 4H). This again means that increased Ki67 expression could be the primary force driving tumor cell invasion. (8) When a big tumor nest produces smaller seeding tumor nests, high Ki67 expression is observed at the conjunction between the two tumor nests (Fig 4I). This indicates that Ki67 could promote the formation of new tumor nests.

### Survival analysis between Ki67 grade or Ki67/CK grade and 5-DFS

**Fig 5:**
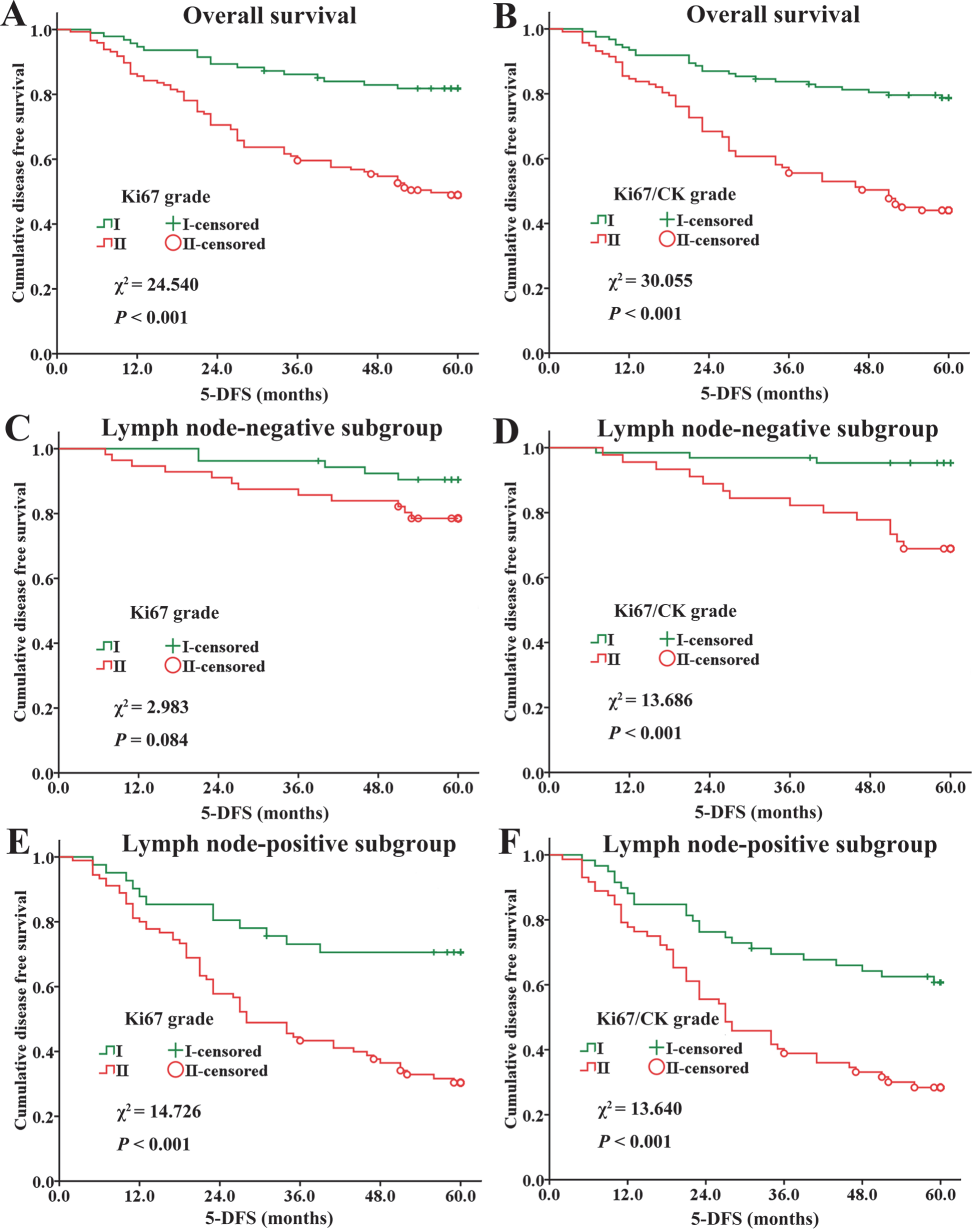
The relationship between Ki67 grade or Ki67/CK grade and 5-DFS in BC patients. Ki67 grade (A) and Ki67/CK grade (B) are negatively related with 5-DFS in all patients; Ki67 grade (C) has no statistical significance in lymph node-negative subgroup, but Ki67/CK grade (D) is negatively related with 5-DFS in lymph node-negative subgroup; Ki67 grade (E) and Ki67/CK grade (F) are negatively related with 5-DFS in node-positive subgroup. BC: breast cancer; 5-DFS: 5-year-disease free survival; CK: cytokeratin.

Survival analysis was performed to investigate the relationship between Ki67 grade or Ki67/CK grade and 5-DFS of BC patients. As shown by the Kaplan-Meier survival curve (Fig 5), Ki67 grade was negatively related with 5-DFS in overall patients (Fig 5A, *P* < 0.001) and lymph node-positive subgroup (Fig 5E, *P* < 0.001), but not in lymph node-negative subgroup (Fig 5C, *P* = 0.084). Ki67/CK grade was negatively related with 5-DFS in overall patients (Fig 5B, *P* < 0.001), lymph node-negative subgroup (Fig 5D, *P* < 0.001) and lymph node-positive subgroup (Fig 5F, *P* < 0.001). This indicates that Ki67/CK grade could be a more suitable parameter to predict the 5-DFS of BC patients.

### Multivariate analysis of Ki67 grade, Ki67/CK grade and traditional parameters

To further validate the prognostic significances of these new parameters, Ki67 grade or Ki67/CK grade and traditional prognostic parameters that had been verified by Kaplan-Meier survival analysis were subjected to Cox proportional hazards model analysis. In Ki67 grade subgroup, multivariate analysis identified 3 variables including Ki67 grade (*P* = 0.047), N stage (*P* < 0.001) and histological grade (*P* < 0.001), as independent prognostic factors for 5-DFS; but HER2 gene (*P* = 0.075), T stage (*P* = 0.365) and ER status (*P* = 0.584) were not independent prognostic factors. In Ki67/CK grade subgroup, multivariate analysis identified 4 variables including Ki67/CK grade (*P* = 0.004), N stage (*P* < 0.001), histological grade (*P* < 0.001), and HER2 gene (*P* = 0.021) as independent prognostic factors for 5-DFS; but T stage (*P* = 0.301) and ER status (*P* = 0.587) were not independent prognostic factors (Table 3).

**Table 3:** Multivariate analysis on major traditional pathological parameters by Ki67 grade or Ki67/CK grade.

Parameters	Analysis by Ki67 grade		Analysis by Ki67/CK grade	
	HR (95%CI)	***P*** value	HR (95%CI)	***P*** value
**T stage**	1.194 (0.814–1.750)	0.365	1.223 (0.835–1.789)	0.301
**N stage**	1.753 (1.446–2. 125)	**< 0.001**	1.819 (1.501–2.204)	**< 0.001**
**Histological grade**	2.958 (1.957–4.472)	**< 0.001**	2.894 (1.924–4.352)	**< 0.001**
**ER status**	0.900 (0. 617–1.312)	0.584	0.902 (0.622–1.308)	0.587
**HER2 gene**	1.553 (0.957–2.522)	0.075	1.748 (1.087–2.812)	**0.021**
**Ki67 grade**	1.773 (1.009–3.117)	**0.047**	ND	
**Ki67/CK grade**	ND		2.019 (1.254–3.251)	**0.004**

ND: not done.

Ki67 grade and Ki67/CK grade were also compared with currently accepted histopathological prognostic factors. As shown in Table 3, the hazard ratio (HR) of Ki67/CK grade (HR 2.019, 95%CI [1.254–3.251]) was higher than N stage (HR 1.819, 95%CI [1.501–2.204]) and HER2 gene (HR 1.748, 95%CI [1.087–2.812]), but lower than the histological grade (HR 3.370, 95%CI [1.125–5.364]). Moreover, the significance and HR of Ki67/CK grade (*P* = 0.004; HR 2.019, 95%CI [1.254–3.251]) was higher than Ki67 grade (*P* = 0.047; HR 1.773, 95%CI [1.009–3.117]). This again indicates, that from the view point of biomedical statistics, Ki67/CK grade could be a better parameter than Ki67 per se for predicting clinical prognosis.

### ROC analysis on independent prognostic factors

**Fig 6:**
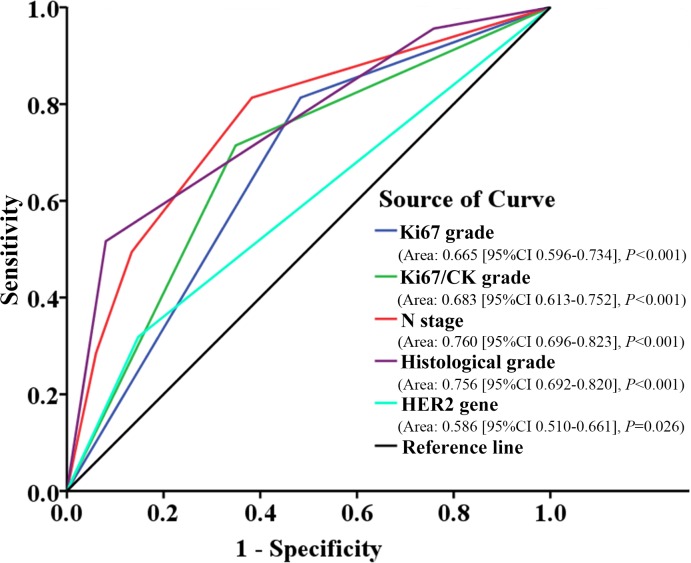
ROC analysis on independent prognostic factors of BC. Ki67/CK grade has higher prognostic value on recurrence than Ki67 grade and HER2 gene, but lower than N stage and histological grade. BC: breast cancer; CK: cytokeratin; N: node; HER2: human epidermal growth factor receptor-2.

Independent prognostic factors verified by multivariate analysis were further studied by ROC analysis to assess their prognostic values. Most of the adopted factors predicted recurrence risk with good performance (*P* < 0.05). In terms of recurrence or not, the prognostic value of Ki67/CK grade (AUC: 0.683, 95%CI: 0.613–0.752, *P* < 0.001) was higher than Ki67 grade (AUC: 0.665, 95%CI: 0.596–0.734, *P* < 0.001) and HER2 gene (AUC: 0.586, 95%CI: 0.510–0.661, *P* = 0.026), but lower than N stage (AUC: 0.760, 95%CI: 0.696–0.823, *P* < 0.001) and histological grade (AUC: 0.756, 95%CI: 0.692–0.820, *P* < 0.001) (Fig 6).

## Discussion

Currently, node-negative BC has been the main part of BC patients [35]. So, more information on BC invasive behaviors will be extracted only from the tumor mass [23]. As an important indicator for tumor cell proliferation, Ki67 has attracted much attention in recent years and proved to be a poor prognosis factor [7,31,36]. Measurement and interpretation of Ki67, however, remain a tremendous challenge for oncologists and pathologists [8,37].

There are three mainstream methods to evaluate Ki67 expression in clinical practice. One is manual counting of Ki67 positive cells in as many as 1,000 tumor cells at the invasive edge, which is the endorsed and frequently used method in clinical practice [17,38–40]. The second method is visual estimation (also known as “eyeballing”), which is a rapid scanning and rough estimation of the Ki67 positivity to produce a global subjective Ki67-positive percentage [9]. This simple and quick method has been reported to have equal if not better reproducibility with the 1,000-cell-counting method [41,42]. However, these two manual counting methods are highly experience-based and subjective. Moreover, both the intra- and inter-observer variations among the experienced diagnostic pathologists are also major obstacles to a harmonized interpretation of the same tissue section. An international Ki67 reproducibility study on BC from the world’s most experienced laboratories revealed such substantial variations in Ki67 scoring among the participating laboratories [39], which reflects the innate technical drawbacks of the manual methodology. The third method is automatic counting by computer software which may be a promising alternative method for Ki67 scoring. The results obtained by automatic counting with traditional computer software is the Ki67 sum [18]. Although this pathologist-assisted computer recognition makes the task more objective and efficient, the final result presented, the Ki67 sum, could not objectively reflect the true biological impact of Ki67 in the specific case studied, because the Ki67 sum is influenced by the number of cancer cells in the analytic fields.

In order to address these disadvantages, many efforts have been made in recent years. Gudlaugsson et al [9] used a software to automatically and simultaneously recognize Ki67 positive and negative cancer cell nuclei in IHC tissue sections. Ki67 Percentage was calculated based on the area of the Ki67 positive and negative cancer cell nuclei. The results indicated that automatic counting on Ki67 was more reproducible and had stronger prognostic value than manual counting. In a recent study by Laurinavicius et al [43], an automatic software with proper validation, calibration and measurement error correction procedures was used to calculate the Ki67 percentage in BC by recognizing Ki67 positive and negative cancer cells, which can increase the accuracy of Ki67 measurement compared to visual evaluation. Similar studies also demonstrated that the consistency between automatic counting and manual counting was acceptable [44,45]. This is a remarkable progress in automatic and objective analysis on Ki67 expression in tumor tissues. However, a common limitation in those studies is that recognition on Ki67 positive and negative cancer cell nuclei was only based on the features of cell nuclei such as color, shape and size, which may be difficult to exclude stroma cells (lymphocyte cells, fibroblast cells, macrophage cells, etc.). Therefore, the accuracy in recognizing Ki67 positive and negative cancer cells nuclei in complex tumor sections is questioned, especially for Ki67 negative cancer cells.

Our previous studies [30,46] showed QDs-based multiple imaging could help predict tumor prognosis and improve tumor classifications. And, QDs-based imaging showed good correlation and consistency with conventional IHC studies, with better image quality and sensitivity [30,46,47]. This study developed a new evaluation method based on advantage of QDs multiple imaging to detect and measure Ki67 expression in BC tissues. Three improvements have been made in this new approach. First, by the QDs multiple imaging technique, the specific Ki67 expression in tumor cell nuclei and CK expression in tumor cell cytoplasm are simultaneously revealed. This clearly defines the areas of interest for study by specifically label CK as previous mentioned [23] with good specificity and clarity. Second, the CRi Nuance multispectral imaging system guarantee that the information simultaneously obtained is the spectral signals of both Ki67 and CK, rather than color signals, further eliminating the interference of noisy signals with similar color but different spectrum. Third, a new parameter, Ki67/CK ratio, has been generated to replace of Ki67 sum or Ki67 percentage. This makes the final result interpretation simpler, easier and more accurate.

Based on this new approach, we systematically investigated the clinical significance of Ki67/CK ratio and Ki67 sum in the context of currently well-recognized histo-pathological prognostic factors, with 5-DFS and clinical cancer recurrence as the primary endpoint. Ki67/CK ratio and Ki67 sum were divided into two grades by X-tile software based on the best *P* value principle, as this method could help distinguish studied subjects into statistically and clinically different subgroups with optimal validity [34]. By this data processing, the Ki67 sum and Ki67/CK ratio were divided into Ki67 grade and Ki67/CK grade, respectively, which were incorporated into the multivariate Cox proportional hazards model analysis. The results demonstrated that both Ki67 grade and Ki67/CK grade had good correlation with patients’ major prognostic factors, and they could divide all BC patients into two subgroups with different 5-DFS as validated by Kaplan-Meier analysis. However, Ki67/CK grade had better distinguishing power than Ki67 grade. While Ki67 grade had statistical significance only in lymph node-positive subgroup but not in lymph node-negative subgroup, Ki67/CK grade achieved statistical significance in both lymph node-positive and lymph node-negative subgroups (Fig 5). In term of predictive performance on 5-DFS, Ki67/CK grade had higher HR than Ki67 grade (2.019 *VS*. 1.773) (Table 3). Moreover, while Ki67/CK grade had correlation with 3 major traditional prognostic factors including N stage, histological grade and HER2 gene, Ki67 grade was only correlated with 2 major traditional prognostic factors. Finally, ROC analysis showed that Ki67/CK grade had higher prognostic value on recurrence than Ki67 grade (Fig 6). All these results provide convincing evidence that Ki67/CK grade could be a better prognostic factor than Ki67 grade, because it has better distinguishing power among BC subgroups, higher HR for 5-DFS prediction, better predicting performance for recurrence and good correlation with more currently accepted pathological prognosticators.

While this study demonstrated the potential that QDs-based nano-pathology could help improve the diagnostic pathology in clinical setting, some limitations in this study should also be acknowledged. First, this study evaluated the relationship between Ki67 expression and patient prognosis by 5-DFS rather than overall survival. As DFS sometimes could not be linearly correlate with overall survival, the clinical impact of Ki67/CK grade on overall survival remains a subject for future study. Second, the method used in this study could not eliminate the interference caused by Ki67 expressed in the stroma, although such Ki67 expression in the stroma is very little. Third, although the construction of TMAs followed strict criteria that only tumor tissues at the cancer invasion edge were selected, not every core of TMAs could completely represent the whole tumor, [23] leading to the possibility of sample selection bias. Therefore, the results from this study should be interpreted with caution and more validation works with larger sample size and longer clinical follow-up are warranted.

## Conclusion

Using QDs-based multiple molecular imaging technology to evaluate Ki67 and CK expression in BC tissues, this study demonstrated that Ki67/CK grade may be better than Ki67 grade in predicting BC prognosis.

## Supporting Information

S1 Dataset Detailed information on Ki67 quantificational value, CK quantificational value, Ki67/CK ratio and clinic-pathological characteristics. (XLS)
